# Supplementary material for: Pyruvate Dehydrogenase Kinase 1 inhibition mediated oxidative phosphorylation enhancement in cartilage promotes osteoarthritis progression
Source: BMC Musculoskelet Disord. 2023 Jul 20;24:597. doi: 10.1186/s12891-023-06585-6 (PMC10357736; doi:10.1186/s12891-023-06585-6)
Supplement: Supplementary file 1 — Additional file 1. [file 12891_2023_6585_MOESM1_ESM.zip › KEGG.Gsea.1653622432464/KEGG_N_GLYCAN_BIOSYNTHESIS.html]

Details for gene set KEGG\_N\_GLYCAN\_BIOSYNTHESIS[GSEA]

|  || Dataset | 55457\_collapsed\_to\_symbols.cls.cls #OA\_versus\_normal.cls.cls #OA\_versus\_normal\_repos |
| Phenotype | cls.cls#OA\_versus\_normal\_repos |
| Upregulated in class | OA |
| GeneSet | KEGG\_N\_GLYCAN\_BIOSYNTHESIS |
| Enrichment Score (ES) | 0.42720807 |
| Normalized Enrichment Score (NES) | 1.5976284 |
| Nominal p-value | 0.012468828 |
| FDR q-value | 0.039553043 |
| FWER p-Value | 0.549 |
Table: GSEA Results Summary

  

Fig 1: Enrichment plot: KEGG\_N\_GLYCAN\_BIOSYNTHESIS      
 Profile of the Running ES Score & Positions of GeneSet Members on the Rank Ordered List

  

| SYMBOL | TITLE | RANK IN GENE LIST | RANK METRIC SCORE | RUNNING ES | CORE ENRICHMENT || 1 | DDOST | dolichyl-diphosphooligosaccharide--protein glycosyltransferase non-catalytic subunit [Source:HGNC Symbol;Acc:HGNC:2728] | 428 | 0.695 | 0.0273 | Yes |
| 2 | MGAT4A | "alpha-1,3-mannosyl-glycoprotein 4-beta-N-acetylglucosaminyltransferase A [Source:HGNC Symbol;Acc:HGNC:7047]" | 657 | 0.604 | 0.0622 | Yes |
| 3 | ALG6 | "ALG6 alpha-1,3-glucosyltransferase [Source:HGNC Symbol;Acc:HGNC:23157]" | 696 | 0.595 | 0.1111 | Yes |
| 4 | DAD1 | defender against cell death 1 [Source:HGNC Symbol;Acc:HGNC:2664] | 737 | 0.585 | 0.1590 | Yes |
| 5 | ALG8 | "ALG8 alpha-1,3-glucosyltransferase [Source:HGNC Symbol;Acc:HGNC:23161]" | 900 | 0.543 | 0.1937 | Yes |
| 6 | ALG5 | ALG5 dolichyl-phosphate beta-glucosyltransferase [Source:HGNC Symbol;Acc:HGNC:20266] | 945 | 0.529 | 0.2364 | Yes |
| 7 | MAN2A1 | mannosidase alpha class 2A member 1 [Source:HGNC Symbol;Acc:HGNC:6824] | 1069 | 0.501 | 0.2705 | Yes |
| 8 | GANAB | glucosidase II alpha subunit [Source:HGNC Symbol;Acc:HGNC:4138] | 1267 | 0.464 | 0.2957 | Yes |
| 9 | MAN1C1 | mannosidase alpha class 1C member 1 [Source:HGNC Symbol;Acc:HGNC:19080] | 1275 | 0.463 | 0.3355 | Yes |
| 10 | RPN2 | ribophorin II [Source:HGNC Symbol;Acc:HGNC:10382] | 1294 | 0.459 | 0.3741 | Yes |
| 11 | ALG9 | "ALG9 alpha-1,2-mannosyltransferase [Source:HGNC Symbol;Acc:HGNC:15672]" | 1487 | 0.431 | 0.3967 | Yes |
| 12 | FUT8 | fucosyltransferase 8 [Source:HGNC Symbol;Acc:HGNC:4019] | 1565 | 0.418 | 0.4272 | Yes |
| 13 | DPM3 | "dolichyl-phosphate mannosyltransferase subunit 3, regulatory [Source:HGNC Symbol;Acc:HGNC:3007]" | 2618 | 0.289 | 0.3706 | No |
| 14 | MAN1B1 | mannosidase alpha class 1B member 1 [Source:HGNC Symbol;Acc:HGNC:6823] | 3447 | 0.207 | 0.3243 | No |
| 15 | ST6GAL1 | "ST6 beta-galactoside alpha-2,6-sialyltransferase 1 [Source:HGNC Symbol;Acc:HGNC:10860]" | 3502 | 0.202 | 0.3377 | No |
| 16 | ALG12 | "ALG12 alpha-1,6-mannosyltransferase [Source:HGNC Symbol;Acc:HGNC:19358]" | 3723 | 0.183 | 0.3366 | No |
| 17 | TUSC3 | tumor suppressor candidate 3 [Source:HGNC Symbol;Acc:HGNC:30242] | 3940 | 0.161 | 0.3339 | No |
| 18 | ALG3 | "ALG3 alpha-1,3- mannosyltransferase [Source:HGNC Symbol;Acc:HGNC:23056]" | 4096 | 0.146 | 0.3346 | No |
| 19 | MAN1A2 | mannosidase alpha class 1A member 2 [Source:HGNC Symbol;Acc:HGNC:6822] | 4272 | 0.131 | 0.3324 | No |
| 20 | STT3A | STT3 oligosaccharyltransferase complex catalytic subunit A [Source:HGNC Symbol;Acc:HGNC:6172] | 4618 | 0.103 | 0.3146 | No |
| 21 | DPM2 | "dolichyl-phosphate mannosyltransferase subunit 2, regulatory [Source:HGNC Symbol;Acc:HGNC:3006]" | 4862 | 0.083 | 0.3029 | No |
| 22 | MGAT5 | "alpha-1,6-mannosylglycoprotein 6-beta-N-acetylglucosaminyltransferase [Source:HGNC Symbol;Acc:HGNC:7049]" | 5086 | 0.066 | 0.2912 | No |
| 23 | MGAT2 | "alpha-1,6-mannosyl-glycoprotein 2-beta-N-acetylglucosaminyltransferase [Source:HGNC Symbol;Acc:HGNC:7045]" | 5317 | 0.046 | 0.2774 | No |
| 24 | DOLPP1 | dolichyldiphosphatase 1 [Source:HGNC Symbol;Acc:HGNC:29565] | 5369 | 0.043 | 0.2772 | No |
| 25 | MGAT1 | "alpha-1,3-mannosyl-glycoprotein 2-beta-N-acetylglucosaminyltransferase [Source:HGNC Symbol;Acc:HGNC:7044]" | 5407 | 0.040 | 0.2777 | No |
| 26 | MGAT4B | "alpha-1,3-mannosyl-glycoprotein 4-beta-N-acetylglucosaminyltransferase B [Source:HGNC Symbol;Acc:HGNC:7048]" | 5956 | -0.002 | 0.2353 | No |
| 27 | RPN1 | ribophorin I [Source:HGNC Symbol;Acc:HGNC:10381] | 6618 | -0.051 | 0.1883 | No |
| 28 | DPAGT1 | dolichyl-phosphate N-acetylglucosaminephosphotransferase 1 [Source:HGNC Symbol;Acc:HGNC:2995] | 7452 | -0.115 | 0.1336 | No |
| 29 | DPM1 | "dolichyl-phosphate mannosyltransferase subunit 1, catalytic [Source:HGNC Symbol;Acc:HGNC:3005]" | 7723 | -0.135 | 0.1244 | No |
| 30 | MGAT3 | "beta-1,4-mannosyl-glycoprotein 4-beta-N-acetylglucosaminyltransferase [Source:HGNC Symbol;Acc:HGNC:7046]" | 8920 | -0.226 | 0.0511 | No |
| 31 | ALG13 | ALG13 UDP-N-acetylglucosaminyltransferase subunit [Source:HGNC Symbol;Acc:HGNC:30881] | 9444 | -0.268 | 0.0338 | No |
| 32 | MAN1A1 | mannosidase alpha class 1A member 1 [Source:HGNC Symbol;Acc:HGNC:6821] | 10207 | -0.341 | 0.0043 | No |
| 33 | B4GALT2 | "beta-1,4-galactosyltransferase 2 [Source:HGNC Symbol;Acc:HGNC:925]" | 10490 | -0.372 | 0.0148 | No |
| 34 | MOGS | mannosyl-oligosaccharide glucosidase [Source:HGNC Symbol;Acc:HGNC:24862] | 10630 | -0.386 | 0.0376 | No |
| 35 | B4GALT1 | "beta-1,4-galactosyltransferase 1 [Source:HGNC Symbol;Acc:HGNC:924]" | 11530 | -0.505 | 0.0118 | No |
| 36 | B4GALT3 | "beta-1,4-galactosyltransferase 3 [Source:HGNC Symbol;Acc:HGNC:926]" | 11748 | -0.540 | 0.0420 | No |
| 37 | MAN2A2 | mannosidase alpha class 2A member 2 [Source:HGNC Symbol;Acc:HGNC:6825] | 11772 | -0.545 | 0.0877 | No |
Table: GSEA details [plain text format]

  

Fig 2: KEGG\_N\_GLYCAN\_BIOSYNTHESIS      
 Blue-Pink O' Gram in the Space of the Analyzed GeneSet

  

Fig 3: KEGG\_N\_GLYCAN\_BIOSYNTHESIS: Random ES distribution      
 Gene set null distribution of ES for **KEGG\_N\_GLYCAN\_BIOSYNTHESIS**
